# "On our own, we can't manage": experiences with infant feeding recommendations among Malawian mothers living with HIV

**DOI:** 10.1186/1746-4358-5-15

**Published:** 2010-10-26

**Authors:** Jennifer M Levy, Aimee L Webb, Daniel W Sellen

**Affiliations:** 1 Department of Anthropology, University of Toronto, 19 Russell Street, Toronto, Ontario, M5S 2S2, Canada

## Abstract

**Background:**

Infant feeding in communities with a high prevalence of HIV and AIDS is a potential challenge for mothers who must ultimately decide how to feed their infants within contexts that constrain their choices.

**Methods:**

We investigated how infant feeding policy recommendations translate into maternal infant feeding decisions and practices using ethnographic research conducted between August 2004 and June 2005 among women participating in a prevention of mother-to-child transmission (PMTCT) program in Lilongwe, Malawi.

**Results:**

Qualitative findings are that maternal ability to adhere to recommendations to breastfeed exclusively for the first six months of infant life was constrained by expectations and psycho-social support. The most salient were women's pre-existing views on breastfeeding, their understanding of the medico-scientific information, and the quality of counselling received. In contrast, maternal decisions to wean were largely influenced by household economic factors and food insecurity.

**Conclusions:**

We conclude that PMTCT programs delivered in ways which "download" the responsibility of adhering to recommendations to women in the absence of adequate psycho-social and livelihood supports contribute to substantial maternal psychosocial distress in this and, likely, similar settings.

## Background

How to design programs to better support infant feeding in HIV-prevalent communities is a challenge for health care practitioners, policy-makers, and scientists, but it is usually women care givers living under critical resource constraints who must ultimately decide among limited choices on how to feed their infants. In this paper, we draw on ethnographic research in a prevention of mother-to-child transmission (PMTCT) program in Lilongwe, Malawi to investigate how infant feeding policy recommendations play out on the ground in mothers' infant feeding decisions and practices. We document and summarize mothers' experiences in mobilizing two components of the infant feeding advice that they received from PMTCT program staff: exclusive breastfeeding to six months and rapid weaning (i.e. the complete cessation of breastfeeding) at six months.

The burden of the AIDS epidemic is greatest in resource poor settings; in particular 22.5 million of the 33.2 million individuals infected with HIV globally live in Sub-Saharan Africa (SSA) [1]. Women bear a disproportionate share of the burden and this, in turn, increases risks of paediatric infection. It is estimated that in 2006 women accounted for 61% of infections in SSA and that 20-30% of pregnant women in the region were living with HIV [2]. Without appropriate interventions, including antiretroviral drugs and exclusive replacement feeding, 30-50% of women will pass the virus to their infants; more than a third of all transmissions will occur during breastfeeding [3-6].

The most recent consensus statement released by the WHO and UNICEF recommends that in settings where replacement feeding can be guaranteed to meet the criteria of affordable, feasible, acceptable, sustainable, and safe (AFASS), HIV positive mothers should exclusively replacement feed their infants from birth [7]. In settings where these AFASS criteria cannot be guaranteed, mothers should exclusively breastfeed their children for the first six months, defined as the provision of breast milk only, with no food, drink, or water given, with the exception of vitamins, minerals, and prescribed medicines. Continued breastfeeding beyond six months is recommended until replacement foods that meet the AFASS criteria become available.

At six months, exclusive breastfeeding no longer satisfies the nutritional demands of the growing infant and mixed feeding, giving breast milk in addition to other foods, becomes necessary. Mixed feeding, however, is associated with increased risk of HIV transmission [3,8-11]. Until recently, it was assumed that exclusive breastfeeding to six months followed by rapid weaning may balance the nutritional and immunological needs of infants against the risk of vertical HIV transmission [12]. Therefore, local policy in many settings in SSA has been to recommend exclusive breastfeeding to six months followed by rapid weaning. Given that new data indicate significant risks of rapid early weaning that more than outweigh the benefits in PMTCT [13-15], there is a pressing need to evaluate the intended and unintended consequences of the implementation of these policies through examination of mothers' experience with programs designed to deliver messages about these recommendations to caregivers.

## Methods

Participants were recruited from a PMTCT program in Lilongwe, which offered routine HIV testing to all pregnant women attending antenatal care. The program reports that uptake of HIV testing was high (82%); however, there was a high rate of loss to follow up among women with positive HIV test results. Less than half of those who tested positive for HIV received the PMTCT program components, which included prophylaxis single-dose nevirapine (sdNVP) for the woman and infant, infant feeding counselling, and postnatal peer support. The World Food Program provided a small monthly ration of maize meal and cooking oil. During the time period of this study, PMTCT program nurses counselled women to either exclusively breastfeed to six months and then rapidly wean their infants or exclusively formula feed from birth. The exclusive breastfeeding option was emphasized as most feasible for this population.

Data collection began in August, 2004 and continued to June, 2005. We conducted ethnographic research using four qualitative methods: (1) participant observation at a government health centre and non-government run PMTCT program; (2) semi-structured interviews and (3) focus groups with women living with HIV; and (4) key informant interviews.

Thirty-four women participated in sequential semi-structured interviews from immediately following their diagnosis through their postnatal care (one to seven interviews each). Women recruited for these interviews were pregnant, had been diagnosed with HIV, and were accessing prenatal care and PMTCT services from the study site. Twenty-one women participated in a series of four focus group discussions in groups of four to six women each. These women had already given birth and were attending a PMTCT program postnatal support group. Twenty-one contextual interviews were conducted with PMTCT nurses (n = 6), government maternal and child health nurses (n = 3), the government medical assistant and clinical officer (n = 2), PMTCT program leaders (n = 3), government maternal and child health policy makers (n = 3), and stakeholders in multilateral and bilateral aid agencies (n = 4). This paper primarily focuses on findings from the interviews and focus groups with women living with HIV and participating in the PMTCT program. Some of the findings from the contextual interviews are presented elsewhere [16,17] and further analysis is planned.

Interview and focus group responses were translated, transcribed, and subjected to thematic content analysis. The emergent themes were analysed using a conceptual framework that sought to identify and interpret evidence of the lived experience of the constraints of decision making in the domain of infant care and feeding. All research participants were fully informed of the study and consented to participate. To assure anonymity of participants, all names used in this paper are pseudonyms. This study was approved by McMaster University Canada's Research Ethics Board, Malawi's National Health Sciences Research Committee, and the Lilongwe District Health Office.

Participants in interviews and focus groups were 18-33 years old and had on average 5.5 years of education. All described themselves as married; 11 were in their second marriage while two were in their third. Women first sought antenatal care for their most recent pregnancy at a mean of 5.5 months gestations (3-8 months range). Of the individually interviewed women, 32 of 34 learned of their HIV status through the PMTCT program.

## Results

Several key themes emerged as supports for and constraints to mothers' ability to mobilize the two key components of the infant feeding advice: exclusive breastfeeding to six months and rapid weaning at six months.

### Confusions about exclusive breastfeeding

All mothers in this study initially chose to breastfeed rather than replacement feed their infants. Several expressed the sentiment that "breast milk is best for the baby". For two mothers who were unable to breastfeed because of breast health problems and a perception of insufficient milk, replacement feeding was understood as a less than ideal way to feed their infants in the early months of life.

Many mothers in interviews and focus groups emphasized the importance of exclusive breastfeeding for the first six months. One participant described the "naturalness" of exclusive breastfeeding: "That is how it is supposed to be. Formula milk is not very good for the baby until six months, when you can even give the baby porridge and other things" (Prisca, Interview 3). Most mothers responded that they chose to exclusively breastfeed, because "that's the advice they give to HIV positive women". Despite this overwhelming support for exclusive breastfeeding, most mothers when probed reported practicing mixed feeding during the first six months postpartum. Multiple factors contributed to mixed feeding, including traditional feeding practices, unclear understandings of what exclusive breastfeeding entails, poor communication on the rationale for the advice, and unclear medico-scientific information.

Mothers reported traditional perceptions of infant gut physiology and a perceived need to stretch or prepare the infant gut.

Interviewer: "Why do you give water?"

Participant: "So that the intestines can open up, I give her warm water...That is to prepare her for the future. Because, if the baby doesn't take water now, then when she grows she will be refusing even to receive porridge." (Joyce, Interview 4)

It was also common for mothers to give their babies gripe water to relieve what was perceived to be babies' stomach pain. Mothers who reported giving their babies water, gripe water, and other foods in addition to breast milk did not necessarily perceive these practices to be in conflict with the practice of exclusive breastfeeding. Rather, these mothers believed themselves to be exclusively breastfeeding and thus reducing the risk of HIV transmission.

Some mothers lacked understanding of the importance of exclusive breastfeeding, which contributed to their feeding practices. "They just told me to exclusively breastfeed for six months, but they didn't tell me the reason why" (Alice, Interview 4). When mothers are not armed with a convincing rationale, they may be less empowered to act on recommendations. One mother described her experience:

"When I delivered, they told me that I shouldn't give my child anything apart from breast milk until I wean him. But I didn't understand the reason why. So sometimes, when the baby was crying, I used to buy him yogurt. Then one day when I went there [PMTCT program] and they asked me, why is your baby growing fat like that? I told them that it's breast milk and yogurt. So they told me to stop giving yogurt. But, again, I didn't know the reason why. So, this other day, it was a certain nurse who told me that my child has sores in the mouth because of the yogurt and in the milk there is HIV. So, if I continue, my child can contract the virus through breast milk and the sores. So I thought they wronged me, because they didn't explain to me very well in the first place. But, luckily enough, when I had him tested for the second time he was negative. But I had worries that I had breastfed him while giving other foods." (Anita, Focus Group 2)

Unclear medico-scientific information regarding the role of exclusive breastfeeding also contributed to confusion and suboptimal infant feeding practices. In this study, mothers knew that the baby could contract HIV through the breast milk. However, the communicated information that exclusive breastfeeding prevents transmission led some women to understand that there is no chance of HIV transmission during the exclusive breastfeeding period. They did not necessarily understand how this is possible, because of the presence of HIV in breast milk. "I keep asking myself that I will breastfeed. Can't the baby catch the virus through the milk? Maybe, in the near future, they will tell me what is going to happen" (Anne, Interview 1).

Perhaps more significantly, many mothers attributed the prevention of HIV during the first six months to prophylactic drugs, rather than to the practice of exclusive breastfeeding. Several mothers commented that the sdNVP that they and their infants received worked to prevent transmission of HIV during the period of exclusive breastfeeding (i.e. that the drug expires at six months) and that is why they are advised to wean the babies at that time. One woman described, "When the mother and child are taking nevirapine you can breastfeed, and the baby will be fine. Yes, there is a chance of transmission, but it's only possible if you exceed six months of breastfeeding. At six months the nevirapine expires" (Esther, Interview 1). Mothers also attributed prevention of HIV during the first six month to co-trimoxazole, an antibiotic given to babies to prevent chest infections.

Participant: "They have given me the medicine which they said can prevent her from being infected. But, after six months, I will follow the advice which I will be told. Because, if I don't, after six months the baby can contract the virus."

Interviewer: "Which medicine is that you were given?"

Participant: "Bactrim [co-trimoxazole]." (Lucy, Interview 4)

Mothers' belief that HIV transmission in the first six months is prevented by drugs may cause them to place less emphasis on the exclusive breastfeeding advice.

While mothers held various views on infant feeding, exclusive or non-exclusive breastfeeding in the first six months was most directly related to their understanding of the communicated infant feeding advice. Mothers who understood how and why they should exclusively breastfeed adhered to this advice. "I am only breastfeeding...Because I was found HIV positive. So, if I give the baby other foods then the baby can contract the virus" (Molly, Interview 3). These mothers frequently described how they viewed their adherence as critical to preventing HIV transmission, and even when probed, stated that they do not give their babies anything other than breast milk.

### The challenge of weaning

Most mothers were determined to wean to avoid transmission, but were unable to within their lived realities of poverty and food insecurity. "We heard the advice that we should stop breastfeeding our babies at six months, and the message is in our ears. But I think that to stop breastfeeding the baby at six months is difficult" (Catherine, Focus Group 1).

However, some mothers struggled with how to balance their belief that breastfeeding is best for infant needs against potential HIV infection. One example is a mother who recognised the existence of two equal and competing health threats posed by the recommendation: potential HIV transmission if she does not wean her infant at six months versus almost certain malnutrition and its detrimental consequences if she does:

"It's difficult to stop breastfeeding the baby when he is still small...They say breast milk is the best food for the baby. Porridge is nothing, so the baby starts swelling because of lack of food." (Nora, Interview 4)

The recognition of this weaning dilemma, however, was unusual amongst the mothers in this study. For most mothers the threat of HIV exceeded that of malnutrition and other weaning complications.

Even in the face of extreme barriers, the determination that mothers had to terminate breastfeeding at six month was evident, as expressed by one woman whose husband had recently died.

Participant: "I am planning to stop breastfeeding when the baby turns six months and introduce her to other foods."

Interviewer: "How do you think that you will manage?"

Participant: "I cannot say I will manage fully/properly, but I will still do it, though with problems."

Interviewer: "What problems are those?"

Participant: "I am saying that, because as of now, I am alone and I cannot manage. But, had it been that my husband was alive, he would have been managing all. But now I have to wait for other people to do things for me, and they cannot manage everything." (Sandra, Interview 5)

Mothers thus desired to at least attempt to follow the advice all the while aware that they might have to return to breastfeeding.

In the context of women's poverty and inadequate food resources, however, the early cessation of breastfeeding was impossible for most mothers in this study despite their initial intentions. The primary constraint to rapid weaning was lack of alternative foods, or funds for such foods, that could sustain their babies after six months in the absence of breast milk. The experiences of mothers whose children had reached six months of age illuminate how unrealistic terminating breastfeeding at six months was for most mothers.

"The decision that I made was to continue breastfeeding my baby, because I couldn't have managed to stop breastfeeding at six months as per the advice, because I didn't have money or support to buy formula milk for my baby." (Meredith, Focus Group 4)

Most mothers discussed a lack of resources to buy formula as their major barrier to terminating breastfeeding. Many mothers chose to delay weaning until they felt the child could survive without breast milk or formula. One mother whose child was fifteen months old described her assessment in relation to the coming harvest, "As for me, I think I am going to stop breastfeeding my baby this coming March, because maize is going to be ready for eating [fresh maize]" (Catherine, Focus Group 1).

Women's various resource capacities thus determined when they were able to stop breastfeeding. Though a few mothers managed to cease breastfeeding at six months, most continued breastfeeding beyond the advised time period. These findings are in contrast to those of Lunney et al, who found that the majority of HIV-infected Zimbabwean mothers, motivated by their fear of transmission, weaned their infants despite similar resource constraints [18]. Women in the current study were convinced that given adequate financial support they could and would terminate breastfeeding immediately at six month. "As for me it's support that I don't have. I can stop breastfeeding my child today, but I will not have food to feed my baby" (Bernadette, Focus Group 3). Mothers' exclusive focus on formula milk suggests that counselling may not properly assist women in assessing locally available and affordable weaning foods, including properly prepared animal milk. Such education is important regardless of the age at which mothers wean.

### The role of stigma

Women were influenced by an expressed cultural norm whereby breastfeeding continues into the third year. However, they said that for them as HIV positive women, it would be better to stop breastfeeding despite the potential for stigma. "Stigma can be there, because people ask a lot of questions about why you are not breastfeeding. But you know how to answer yourself" (Florence, Focus Group 3). Some mothers expressed that protecting their own and child's health was more important than potential stigmatization; similar findings were also reported by Levy and Storeng [19]. Indeed, poverty seemed a greater influence on infant feeding than stigma or specific beliefs around infant feeding.

"I decided [to breastfeed for] six months, but because of the issue of money it is difficult...But it would have been better if they would have an alternative like giving us formula milk then we would stop." (Janice, Focus Group 3)

### The psychosocial burden of downloaded responsibility

When exclusively breastfeeding to six months, mothers worried about the potential risk of HIV transmission to their children. One mother who was exclusively breastfeeding described her emotional responses about her breastfeeding decisions,

"I worry, because I am breastfeeding my baby. And I feel bad that maybe I may infect her with my HIV. And I feel that I am ruining her future and infringing on the baby's rights. Because a baby has the right to grow up and go to school. But, because I am feeding her infected milk, she can die quickly, since another way of transmitting HIV is through breast milk." (Anne, Interview 3)

When weaning, mothers worried about the smallness of their babies and their financial inability to offer adequate replacement foods. Worry and frustration became more severe after six months for those mothers who were unable to cease breastfeeding. One mother described, "We feel bad thinking that, as the baby is being breastfed, he or she can contact the virus in the process" (Isabel, Focus Group 1).

While women themselves felt guilty they also blamed the PMTCT program for the undue burden placed on them. "I feel this advice is very painful, because they are asking us to stop breastfeeding our babies at six months. But we don't have money or milk to give to our babies, so this really is very painful to us" (Janice, Focus Group 3). Women, therefore, related that more assistance was needed to successfully achieve the infant feeding advice. "I think there is unfairness because, if they know that the baby is to stop breast milk, then they should find us an alternative solution. Because, on our own, we can't manage" (Laura, Focus Group 1). To many women, the advice was viewed as inappropriate for their situation. "This advice which we were given by the doctors [nurses] is good, but only somebody who has money can manage to follow the advice, not us" (Meredith, Focus Group 4). Women recognized that better options and further support are critical for weaning: including, cheaper options for formula, food security, income generating programs, and poverty alleviation.

The PMTCT program staff were cognizant of the dilemma and burden of the advice given. The PMTCT program manager described,

"We counsel these women to stop breastfeeding their babies at six months of age. But, if we look, the socioeconomic status of our country, the baby needs to have some supplementary foods after six months. But, at the same time, we counsel that mother to stop breastfeeding. She doesn't have something to give the baby, so some babies also develop malnutrition because they have nothing. So, as a program, the challenge is that we have nothing to supplement these women but, at the same time, we are giving them the information that, if they continue breastfeeding at the same time as supplementary feeding, they increase the chances of HIV infection in the babies. So that's also the main challenge."

## Discussion

The findings can be discussed as they relate to suggested improvements in PMTCT programs related to counselling and support, as well as broader policy recommendations.

### Quality counselling for exclusive breastfeeding

All participants chose initially to breastfeed their infants and respondent statements indicated that breastfeeding was considered the best option for infant feeding by all participants in this study. This finding aligns with recent national data on high uptake of breastfeeding (99%) and a long median duration of 23.2 months in Malawi [20]. Thus, there is evidence that strengthened PMTCT programming can continue to leverage the strong support for breastfeeding expressed by clients.

Many respondents reported mixed feeding, a finding that concords with recent national data showing that exclusive breastfeeding is not the dominant pattern in Malawi [20]. Qualitative analysis suggests that local perceptions of a need to train the infant gut through feeding other substances provided some rationale for this practice, but perhaps even more salient was the apparent misunderstanding and confusion about biological rationale for the guidelines on exclusive breastfeeding. Specifically, the findings demonstrate that mothers' ability to follow the infant feeding recommendation to exclusively breastfeed for six months relates to women's pre-existing views on breastfeeding, the quality of the counselling they received, and their understanding of the medico-scientific information including how infant feeding practices and prophylaxis therapies each can reduce the risk of transmission.

Women trust highly the role of the pharmaceutical intervention, sdNVP, in preventing transmission, and misattribute prevention properties to co-trimoxazole, while simultaneously downplaying the role of exclusive breastfeeding in reducing transmission. Analysis suggests that this relates to the primacy of the biomedical paradigm within the PMTCT program and the corresponding focus on biomedical treatments over women's counselling and socioeconomic needs [17].

Several studies in other settings have shown that when exclusive breastfeeding advice is well defined and women understand the advice well, they succeed in following it [21,22]. Therefore, PMTCT programs need to focus on and more directly guide the content, duration, and quality of infant feeding counselling. In this setting, counselling can be strengthened by addressing women's pre-existing infant feeding beliefs, clearly defining what exclusive breastfeeding entails, and plainly explaining the medico-scientific rationale, as an important component of the advice to prevent inadvertent mixed feeding.

New studies are demonstrating the efficacy of antiretroviral regimes administered during breastfeeding in PMTCT [23]. There is a need to quickly implement new scientific findings on effective antiretroviral therapy into PMTCT policies and programs.

### Support for infant weaning

Unlike exclusive breastfeeding, misunderstandings regarding weaning did not contribute to whether mothers rapidly weaned their infants at six months. Some mothers recognized a dilemma between avoiding HIV transmission by rapidly weaning and their knowledge that the weaning foods available within their financial means were inadequate to support their child's health and nutrition needs. This dilemma around the appropriateness and timeliness of weaning and introduction of complementary foods is described in the literature [24-29] and occurs as the infant makes the necessary transitions from a diet composed exclusively of breast milk to one that includes complementary, often home-produced, foods. If these foods are nutritionally inadequate and/or contaminated with pathogens, as is more common in resource poor settings, they can contribute to infant poor health outcomes including infectious disease morbidity and growth faltering.

For most mothers, however, the decision to delay weaning was driven largely by their low economic capacity - despite wanting to rapidly wean they were financially incapable of sourcing appropriate weaning foods. Studies have shown that increased morbidity, infection, diarrhea, and malnutrition are known potential outcomes of poorly managed weaning, but that these can be avoided through delivery of appropriate and effective counseling, education, and support for weaning [30]. The qualitative findings presented here indicate that within the context of PMTCT, programs need to include mechanisms for livelihood and/or food support of HIV-affected women, in addition to education and counseling. Such mechanisms are required to enable effective and appropriate weaning or continued breastfeeding with adequate complementary feeding, depending on the mother's choice, thereby reducing the risk of potential poor infant health outcomes.

While some studies have indicated stigma as a barrier to achieving recommended infant feeding practices [31-33], women in this study overwhelming downplayed the importance of stigma in their infant feeding decisions. The lived experience of the few mothers who successfully weaned their children captured in this study strongly suggests that financial support rather than stigma or specific beliefs around infant feeding is the greatest barrier. Nevertheless, counselling on strategies for reducing and overcoming stigma would be potentially helpful.

### The psychosocial burden of downloaded responsibility

Infant feeding decisions in the context of HIV proved to be an emotional issue for women in this setting; both the recommendation to exclusively breastfeed to six months and to rapidly wean thereafter created substantial distress among women and was of evident concern to PMTCT program managers. Nonetheless, as is the case with other components of the program, the burden of responsibility of achieving recommended infant feeding advice and thus avoiding HIV transmission is oftentimes downloaded to mothers with the least capacity from international and national health systems and policy contexts that have much greater capacity and potential to offer appropriate and sustainable solutions [16]. This downloading of responsibility causes substantial psychosocial distress. Critically, in women living with HIV, poor psychosocial health has physiological consequences and is associated with more rapid disease progression, increased viral loads, and mortality due to AIDS [34]. Thus, supports to women that address the constraints outlined in this study and others must be a vital component of comprehensive PMTCT programs to maximize the reduction of HIV transmission through breastfeeding and safeguard both women's and their infants' health.

The programmatic findings and recommendations presented here additionally point to the need for more comprehensive policies on infant feeding in the context of HIV. National and international policies that specifically guide infant feeding counselling for HIV positive women during pregnancy, at birth, and postpartum could more effectively reduce vertical transmission. Strengthened policies that better facilitate program design and delivery would likely include minimum requirements on the content, number, and length of counselling sessions, and deployment of effective tools to assess AFASS for infant feeding options and identify locally available weaning foods before advising women to wean. Moreover, the gap between women's resource realities in deciding how to feed their infants and the goal of reducing transmission through breast milk could be bridged by policies that address food insecurity, as a limitation to mother's infant feeding choices. Such policies require a funding commitment for human resources and training to achieve quality counselling, and a broader definition of PMTCT as not exclusively a biomedical problem, but also a psychosocial and economic issue.

## Conclusions

Though it effectively eliminates the risk of HIV transmission via breast milk, exclusive replacement feeding from birth is a difficult option for most Malawian mothers. Women enter the PMTCT program with pre-existing views on the best way to feed their infants; as such, overcoming mothers' challenges to adherence to the infant feeding advice depends on adequate counselling, clear communication of the medico-scientific rationale for the recommended practices, and resource support due to the prohibitive cost of formula and fuel to prepare it correctly. Unfortunately, mothers' experiences in mobilizing infant feeding advice demonstrate that these components are often insufficient. Without adequate education and support, many mothers are unlikely to achieve exclusive breastfeeding for the first six months. Furthermore, resource difficulties in combination with high prevalence of child undernutrition [20,35-37] and the cultural preference for extended breastfeeding may ultimately make rapid weaning inadvisable and unacceptable.

While the revised international guidelines now recommend continued breastfeeding until AFASS replacement foods become available rather than rapid weaning, and new research suggests a potential for maternal postpartum antiretroviral therapies to reduce transmission through breastfeeding, adequate and appropriate supports to women, including education and resources, will remain necessary to ensure that mothers can adequately and safely complementary feed their infants beyond six months so as to avoid infant malnutrition and subsequent morbidities.

## Competing interests

The authors declare that they have no competing interests.

## Authors' contributions

JML designed and conducted the research and transcript analysis, and contributed to data interpretation and manuscript preparation. AW contributed to data interpretation and manuscript preparation. DS contributed to manuscript preparation. All authors read and approved the final manuscript.
